# Wound healing activity of *Tropaeolum tuberosum*-based topical formulations in mice

**DOI:** 10.14202/vetworld.2022.390-396

**Published:** 2022-02-19

**Authors:** Carmen R. Silva-Correa, Greysi I. Pazo-Medina, Víctor E. Villarreal-La Torre, Abhel A. Calderón-Peña, Cinthya L. Aspajo-Villalaz, José L. Cruzado-Razco, Jorge Del Rosario-Chávarri, Anabel D. González-Siccha, Luz M. Guerrero-Espino, María V. González-Blas, William A. Sagástegui-Guarniz, César D. Gamarra-Sánchez, Julio Hilario-Vargas

**Affiliations:** 1 Department of Pharmacology, School of Pharmacy and Biochemistry, National University of Trujillo, Trujillo, Perú; 2 Department of Biological Chemistry and Animal Physiology, School of Biological Science, National University of Trujillo, Trujillo, Perú; 3 Department of Biochemistry, School of Pharmacy and Biochemistry, National University of Trujillo, Trujillo, Peru; 4 Department of Physiology, School of Medicine, National University of Trujillo, Trujillo, Perú

**Keywords:** histology, histopathological changes, skin, topical formulations, *Tropaeolum tuberosum*, wound healing

## Abstract

**Background and Aim:**

*Tropaeolum tuberosum* Ruiz and Pavón, a tuber native to South America, is characterized by its antioxidant, antimicrobial, and anti-inflammatory properties that contribute to wound healing. This study aimed to evaluate the healing effect of the topical *T. tuberosum* formulations (gel and cream) on induced wounds in mice.

**Materials and Methods:**

Here, an acidic ethanolic extract (1.5 N hydrochloric acid and 96% ethanol at the ratio of 15:85, v/v) was prepared with the tubers of *T. tuberosum* ecotype black and incorporated into topical cream and gel formulations at 1%. Thirty-twoBalb/c mice were divided into four experimental groups receiving daily topical treatments for 14 days: Group I (control; no treatment), Group II (a commercial ointment of neomycin, polymyxin B, and bacitracin), Group III (1% *T. tuberosum* gel), and Group IV (1% *T. tuberosum* cream). The wound closure in the mice was determined during the treatment; then, they were euthanized to obtain skin samples for histopathological analysis.

**Results:**

Groups III and IV showed a higher percentage of wound closure from the 6^th^ day of the treatment. From the 8^th^ day, the effect was greater in Group III. The healing effect was confirmed by the histopathological changes.

**Conclusion:**

This study concludes that the topical formulations of *T. tuberosum* demonstrate wound-healing activity in mice, and the most effective treatment is the 1% gel formulation.

## Introduction

Cutaneous wounds are a serious health problem worldwide and are frequently associated with high costs and ineffective treatments [1,2]. They are characterized by a disruption of the cellular and structural integrity of skin tissue layers [3,4], caused by physical, chemical, thermal, microbial, or immunological damage to tissues [5,6]. Wound healing is a complex, dynamic, and integrated process involving three phases, inflammatory, proliferative, and maturation or remodeling [7-9]. Excessive production of reactive oxygen species, which is correlated with oxidative stress, in inflammations and infections prolong the wound healing process [10,11]. Therefore, compounds with antimicrobial, antioxidant, and anti-inflammatory properties will be helpful to wound healing [4,12,13]. The primary goals of wound treatment are rapid wound closure and the formation of a functional and esthetically satisfactory scar [14]. Dressings and topical products, such as creams and gels, are used in clinical practice. However, they are often costly or unsuccessful and have side effects [15,16].

Using medicinal plants and phytotherapeutics are a new strategy for treating burns, cuts, and wounds [1,17]. Phytocompounds or secondary medicinal plant metabolites have an important role in wound healing as they promote natural tissue repair [5,18,19]. For example, tannins are bacterial growth inhibitors, and terpenoids have astringent and antimicrobial properties. In addition, flavonoids and anthocyanins are powerful antioxidants, anti-inflammatory, and antimicrobial phytoconstituents [1,6,20]. These phytocompounds can contribute to healing independently or synergistically [11,21].

*Tropaeolum tuberosum* Ruiz and Pavón, also known as “mashua,” “isaño,” or “cubio,” is a native tuber to South America. It is generally cultivated by farmers in Colombia, Ecuador, Bolivia, and Peru [22,23]. The skin color of *T. tuberosum* tubers can be in different shades of ivory white, yellow, orange, or purple. In traditional medicine, *T. tuberosum* is used to treat kidney diseases, prostatic hyperplasia, wounds, and parasitic infections [22-24]. The metabolites found in *T. tuberosum* include phenolic compounds, carotenoids, glucosinolates, anthocyanidins, proanthocyanidins, flavonols, triterpenes, and flavones [24-28]. Metabolites have become a focus of research in recent years, as they contribute to the antioxidant, anti-inflammatory, and antimicrobial properties of plants [29-32].

Medical plants are a resource for developing new drugs for wound healing that is safer, more effective, and affordable [4,5]. Therefore, there is an urgent need to validate the efficacy of herbal treatments in wound healing and apply them in wound care [17].

Therefore, this study aimed to evaluate the healing effect of the topical *T. tuberosum* formulations (gel and cream) on induced wounds in mice.

## Materials and Methods

### Ethical approval

The study was approved by the Ethics Committee of the Facultad de Farmacia y Bioquímica of the Universidad Nacional de Trujillo with the document COD. N°: P-011-19/CEIFYB.

### Study period and location

The study was conducted from October 2020 to March 2021. All processes were performed in Toxicology Laboratory, School of Pharmacy and Biochemistry, National University of Trujillo**.**

### Biological material

Thirty-two three-and-a-half-month-old male Balb/c mice (Instituto Nacional deSalud, Peru) weighing 35-40 g were used for this research. We selected males only to avoid hormonal influence. All mice were kept in cages and under standard environmental conditions of a photoperiod of 12/12 h dark/light cycles at 25±2°C. They were given a balanced diet, and water was administered *ad libitum*.

### Vegetal material

Tubers of *T. tuberosum* Ruiz and Pav., black ecotype, were collected from the Province of Otuzco, Region La Libertad, Peru. The taxonomic identification was conducted in the *Herbarium Truxillense* – Universidad Nacional de Trujillo (Code No. 59898).

### Chemical composition

A 2.5 mg/mL solution from the dry extract was prepared using methanol. This solution was passed through a 0.22 μmpolytetrafluoroethylene syringe filter before passing through the ultra-performance liquid chromatographyAcquity H-Class Quaternario^®^ system (Burnsville MN, United States). Chromatographic separation was achieved on AcquityHSS C18^®^ column (Burnsville), size: 100×2.1 mm, 1.7 μm. Formic acid 0.1% (v/v) in water (A) and MeCN (B) mobile phases was used. The gradient conditions were as follows: 0.0-4.17 min 3-10% B; 4.17-6.25 min 10-15% B; 6.25-8.34 min 15% B; 8.34-10.42 min 15-20% B; 10.42-14.49 min 20-25% B; 14.49-16.67 min 25-30% B; 14.49-18.76 min 30-50% B; 18.76-21.67 min 50-90% B; and 21.67-25.0 min 90% B. The flow of the mobile phase was 300 μL/min and the injection volume, 10 μL. The column temperature was kept at 40°C.

The triple quadrupole mass spectrometer was equipped with an electrospray ion source operated in positive and negative ionization mode. The “cone voltage” was kept at 40 V. The drying temperature was set at 450°C and the dry gas flow rate was set at 13 L/min. Nitrogen was used as the dry gas, fog gas, and collision gas. The collision energy was set at 30 eV. High-resolution electrospray ionization mass spectrometry and tandem mass spectrometry (MS/MS) spectra were acquired in an m/z range of 50-2000 amu.

### Preparation of the extract

The tubers were washed, cut, and placed in an extraction solution of hydrochloric acid 1.5 N and 96% ethanol (15:85, v/v). Next, the mixture was macerated for 72 h in an amber flask with daily stirring. Then, it was filtration and concentrated in a rotary evaporator at 40°C. Finally, the dry extract was placed in an amber container and stored at −20°C.

### Preparation of topical formulations

The cream was formulated using Lanette wax, stearic acid, solid petroleum jelly, liquid petrolatum, glycerin, propylene glycol, and water. The gel was prepared using Carbopol 940, liquid glycerin, propylene glycol, triethanolamine, and water [33,34]. The extract was incorporated into the cream and gel, respectively until its concentration reached 1%. Finally, the topical formulations were packaged, labeled, and stored atroom temperature (21°C) until use.

### Healing evaluation

First, the back of a mouse was shaved for the wound induction 24 h later. Next, an anesthetic of a 2% lidocaine cream was applied topically, and an approximately 1 cm cut was made perpendicularly to the longitudinal axis of the mouse. Then, the healing was measured using a Vernier caliper [35].

Thirty-two Balb/c mice were randomly divided into four experimental groups with eight mice per group. Group I (control) received no treatment. Group II received a commercial ointment of neomycin, polymyxin B, and bacitracin, Group III received the 1% gel), and Group IV received the 1% cream.

The mice received adaily topical treatment applied with sterile swabs for 14 days. Wound healing was evaluated during the 14-day treatment by measuring the size of wound closure.

Wound healing was measured using a metric Vernier caliper, and the percentage of wound closure was calculated using the following formula:


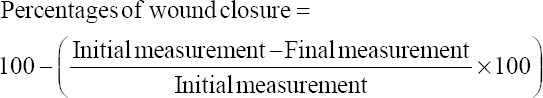


### Histopathological study

The mice were euthanized using sodium pentobarbital at 60 mg/kg/v.ip. Skin samples were obtainedby cutting a tissue 1.5 cm in length and 1 cm in width around the scar. These samples were stored in sterile bottles with 10% formaldehyde for histopathological analysis.

### Statistical analysis

The figures were prepared using R-4.1.1 for Windows^®^ (https://cran.r-project.org/bin/windows/base/), and the data were subjected to analysis of variance followed by the *post hoc* Tukey test. A difference with p<0.05 was considered statistically significant.

## Results and Discussion

**Figure-1:**
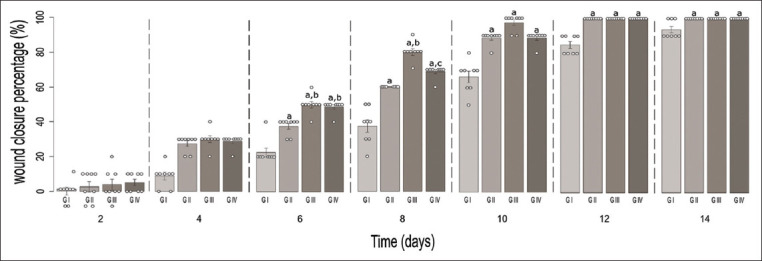
Percentage of wound closure in experimental groups during the treatment days. (a) There is a significant difference with Group I. (b) There is a significant difference with Group II. (c) There is a significant difference with Group III. p<0.05, n=8.

Wound closure is a parameter that indicates the progress in the healing process. The wound healing in the experimental groups during the 14 days treatment was compared (Figure-1). The percentages of wound closure on day 6 in Groups II (commercial ointment), III (1% gel), and IV (1% cream) were significantly different compared with Group I (control). At the same time, Groups III and IV showed a significantly higher percentage of wound closure than Group II (p<0.05). From the 8^th^ day of the treatment, a significant difference in wound closure appeared between the groups receiving the topical *T. tuberosum* formulations; wound closure was greater in Group III, which received the 1% *T. tuberosum* gel. The final evaluation of the treatment on the 14^th^ day revealed that Groups II, III, and IV completed the wound closure compared to Group I (Control).

**Figure-2:**
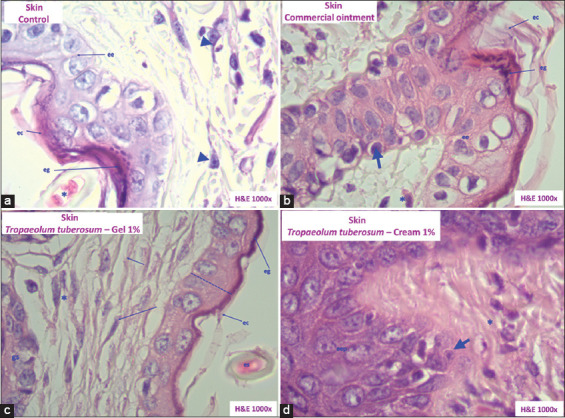
Histopathological sections of the skin of *Mus musculus* Balb/c. (a) Group I (control). (b) Group II (commercial ointment). (c) Group III (1% gel). (d) Group IV (1% cream). Keratinous stratum corneum (ec), stratum granulosum (eg), stratum spinosum (ee), epidermal strata (eep), sweat glands (gs), dermal papillae (arrow), fibroblasts (*), few fibroblasts (arrowheads), epidermis (dashed line) (hematoxylin and eosin stained, 1000×).

Next, the histopathological changes in the mouse skin samples were examined (Figure-2). In Group I, the differentiation of basal cells was not observed in the epidermis, while connective tissues were observed with few fibroblasts arranged in parallel in the dermis. These findings corresponded to progressive and physiological scarring; however, the presence of inflammatory cells resulted from the induced injury. On the other hand, Group II exhibited the differentiation of basal cells, indicative of re-epithelialization. Meanwhile, the connective tissues in the dermis contained fibroblasts, collagen, and elastic fibers to fill and contract the wound, an effect attributable to the healing cream.

The groups that received topical *T. tuberosum* formulations exhibited greater healing activities than Groups I and II. For example, Group III showed a continuous re-epithelialization at the epidermal level. Meanwhile, the gel treatment indicated intense wound healing activity in the dermis, as indicated by the parallel arrangement of fibroblasts and the abundance of collagen. On the other hand, Group IV demonstrated the recovery of the epidermal layers, with corneal, granular, spinous, and cell migration to form the dermal papillae. Meanwhile, in the dermis, abundant collagen and sweat gland activity in tissue replacement were observed, attributable to the effect of *T. tuberosum* cream.

The topical administration of *T. tuberosum* gel and cream significantly increases wound healing in a mouse wound model. The literature suggests that the anti-inflammatory, antimicrobial, and antioxidant properties of *T. tuberosum* may have potential wound healing effects. *T. tuberosum* exerts an anti-inflammatory effect by inhibiting the immune response induced by tumor necrosis factor-alpha and nuclear factor kappa B (NF-kB) [23]; therefore, it likely acts in the inflammatory phase in wound healing that precedes the proliferative and maturation phases [8].

**Figure-3:**
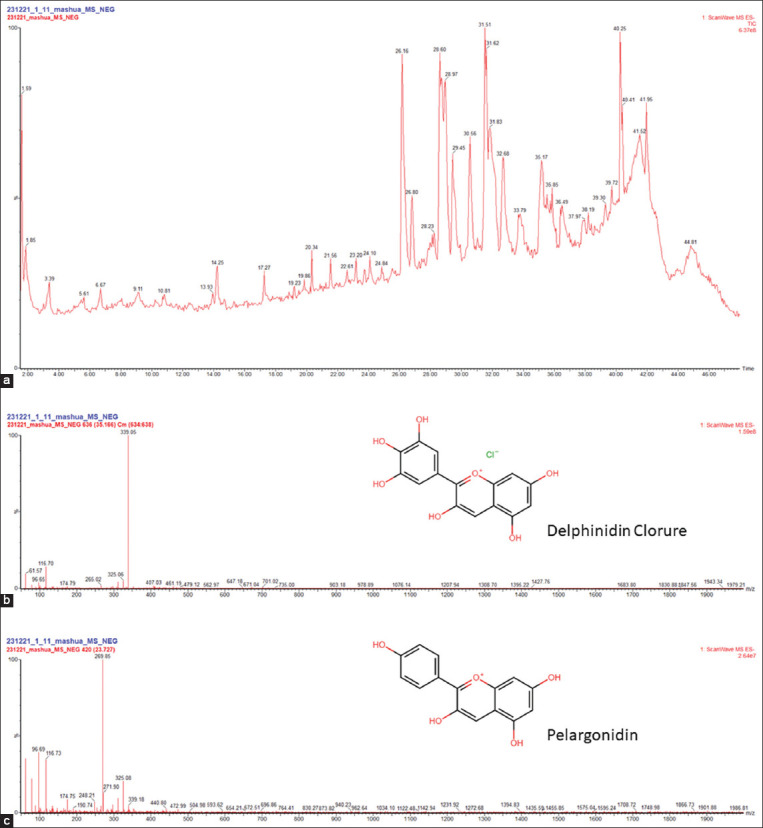
(a) Ultra-performance liquid chromatography-ultraviolet-tandem mass spectrometry (MS/MS) chromatogram from the ethanol extract from mashua. (b) MS/MS spectra obtained m/z 339.05 for delphinidin chloride and (c) m/z 269.85 for pelargonidin.

*T. tuberosum* contains many phenolic compounds and a high antioxidant capacity [34,36]; this property is also related to wound healing. In wounds, there is an increase in reactive oxygen species that promote the inflammatory condition and delay healing [11,37]. The black ecotype of *T. tuberosum* “black mashua” [26] has cyanidin-3-glucoside, an important compound for wound healing [24]. In this study, we also found the *T. tuberosum* extract contained delphinidin and pelargonidin (Figure-3), other anthocyanidins that could be involved in biological activities, such as antioxidant and anti-inflammatory activities [31]. Anthocyanidins are glycosides [28]; however, the use of acidic alcohol could hydrolyze these anthocyanidins in this study. Other compounds were found in *T. tuberosum* extract but could not be identified; however, according to the previous studies, the extract also contains alkamides and glucosinolates, as the hydrolysis of glucosinolates generates macamides and phenolic compounds [23,26].

This study demonstrates that the healing and repair of wounds are accelerated with the topical treatment of *T. tuberosum*. In addition, the histopathological changes in the skin samples from Groups III and IV displayed an increase in cell proliferation and collagen synthesis in the wound. Such histological changes are supported by the presence of newly formed fibroblasts, collagen fibers, and blood vessels [38].

Fibroblasts play a role in wound regeneration and healing and are involved in the production of collagen and other proteins from the extracellular matrix [39]. Collagen deposition is a critical phase in wound healing that provides the matrix for angiogenesis and tissue restoration [40].

When the physiological repair response in the normal healing process is inadequate, there are two main outcomes, an ulcerative skin defect (chronic wound) or excessive scar formation (hypertrophic scar or keloid) [41]. The latter outcome was observed in Group I (control) that presented scars in the epidermis.

A comparison of the effect of *T. tuberosum* extract on Groups III and IV showed that the 1% gel formulation had greater healing activity, resulting in wound closure in less time, consistent with the observed histological characteristics in the mouse skin analysis. The superiority of the gel formulation can be explained by the advantages exhibited by gels or hydrogels, such as providing a conducive, wet environment for the wound area and acting as an appropriate vehicle for the topical administration of plant extracts.

In addition, gel formulations can slowlyrelease active ingredient over time; therefore, they are suitable for wound healing [42]. The *T. tuberosum* extract has many components; in addition, we found that it contained anthocyanins delphinine and pelargonidin, which may be involved as metabolites related to its healing properties. *In vitr*o studies on human fibroblasts showed that anthocyanins increased collagen level [39]. Anthocyanins inhibit the translocation of NF-kB from the cytosol to the nucleus, reducing inflammation during wound healing [43].

## Conclusion

Topical formulations prepared based on the ethanolic extract of *T. tuberosum* Ruiz and Pav. black ecotype can accelerate the healing of the induced wounds in mice. The 1% gel formulation was the most effective treatment. The healing mechanism of *T. tuberosu*m may be related to phytoconstituents, such as anthocyanins and phenolic compounds that exert antioxidant, antimicrobial, and anti-inflammatory effects, which contribute to optimal healing. *T. tuberosum* extract contains many components of research interest, and only two anthocyanins have been investigated; in addition, pharmaceutical formulations have been evaluated. Covid-19 pandemic was a great limitation for the development of this research. The following areas for future research emerged: isolation of the components, evaluation of toxicity studies, preparation of different concentrations and formulations, additional studies in different animal models.

## Authors’ Contributions

WAS: Collected the plant species and entered the herbarium. GIP: Produced the first draft. VEVT and LMG: Prepared cream and gel formulations. CRS, JLC, and ADG: Performed organ harvesting for histopathological analysis. WAS, VEVT, and CRS: Performed the chemical composition by ultra-high-performance liquid chromatography–MS/MS. JDR and JH: Performed the statistical analysis and the preparation of images. AAC and CLA: Kept the animals during the investigation and administered treatments. CDG and MVG: Carried out the preparation of extract. All authors have read and approved the final manuscript.
